# Genome sequence of *Monilinia vaccinii-corymbosi* sheds light on mummy berry disease infection of blueberry and mating type

**DOI:** 10.1093/g3journal/jkaa052

**Published:** 2021-01-04

**Authors:** Ashley G Yow, Yucheng Zhang, Kamaldeep Bansal, Stephen M Eacker, Shawn Sullivan, Ivan Liachko, Marc A Cubeta, Jeffrey A Rollins, Hamid Ashrafi

**Affiliations:** 1 Department of Horticultural Science, North Carolina State University, Raleigh, NC 27695, USA; 2 Department of Plant Pathology, University of Florida, Gainesville, FL 32611, USA; 3 Phase Genomics Inc., Seattle, WA 98109, USA; 4 Department of Entomology and Plant Pathology, Center for Integrated Fungal Research, North Carolina State University, Raleigh, NC 27606, USA

**Keywords:** blueberry, mummy berry, *Monilinia vaccinii-corymbosi*, *Mvc*, disease, blight, pathology, mating-type, effectors

## Abstract

Mummy berry disease, caused by the fungal pathogen *Monilinia vaccinii-corymbosi* (*Mvc*), is one of the most economically important diseases of blueberries in North America. *Mvc* is capable of inducing two separate blighting stages during its life cycle. Infected fruits are rendered mummified and unmarketable. Genomic data for this pathogen is lacking, but could be useful in understanding the reproductive biology of *Mvc* and the mechanisms it deploys to facilitate host infection. In this study, PacBio sequencing and Hi-C interaction data were utilized to create a chromosome-scale reference genome for *Mvc*. The genome comprises nine chromosomes with a total length of 30 Mb, an N50 length of 4.06 Mb, and an average 413X sequence coverage. A total of 9399 gene models were predicted and annotated, and BUSCO analysis revealed that 98% of 1,438 searched conserved eukaryotic genes were present in the predicted gene set. Potential effectors were identified, and the mating-type (*MAT*) locus was characterized. Biotrophic effectors allow the pathogen to avoid recognition by the host plant and evade or mitigate host defense responses during the early stages of fruit infection. Following locule colonization, necrotizing effectors promote the mummification of host tissues. Potential biotrophic effectors utilized by *Mvc* include chorismate mutase for reducing host salicylate and necrotrophic effectors include necrosis-inducing proteins and hydrolytic enzymes for macerating host tissue. The *MAT* locus sequences indicate the potential for homothallism in the reference genome, but a deletion allele of the *MAT* locus, characterized in a second isolate, indicates heterothallism. Further research is needed to verify the roles of individual effectors in virulence and to determine the role of the *MAT* locus in outcrossing and population genotypic diversity.

## Introduction

Biotic stresses, including plant fungal diseases, are among the most significant constraints to crop production (Crous *et al.* 2016). Economic losses due to fungal disease contribute to overall food quality reduction, food waste, and increased production costs (Almeida *et al.* 2019). One approach to understanding and mitigating disease occurrence is to uncover the genetic mechanisms pathogens use to induce disease (Perez-Nadales *et al.* 2014). The genomes of fungal pathogens contain coding sequences for virulence factors, which are required for them to recognize, adhere to, colonize, and acquire nutrients from a host plant (Chibucos and Tyler 2009; Meng *et al.* 2009; Haueisen and Stukenbrock 2016; Bezrutczyk *et al.* 2018). By sequencing the genomes of economically important fungal pathogens, an array of genes encoding important developmental, mating, and virulence proteins have been uncovered (Amselem *et al.* 2011; Chitrampalam *et al.* 2013; Liu *et al.* 2014; Derbyshire *et al.* 2017; Van Kan *et al.* 2017; Rivera *et al*. 2018; Abate *et al.* 2018; De Miccolis Angelini *et al.* 2018). In turn, breeding approaches and fungicide development efforts can be guided by these findings to create highly targeted products for growers (Acero *et al.* 2011; Vleeshouwers and Oliver 2014). Ultimately, these resources lead to more sustainable practices for reducing plant diseases in agricultural systems.

The genus *Monilinia*, within family Sclerotiniaceae, contains ∼30 fungal pathogen species that affect a range of fruit crops, including stone fruits, pome fruits, and berries (Batra 1983; Willetts and Harada 1984; Holst-Jensen *et al.* 1997; Rivera *et al.* 2018). Mummy berry disease, caused by the fungal pathogen *Monilinia vaccinii-corymbosi* (Reade) Honey (hereafter referred to as *Mvc*), is one of the most economically important diseases of blueberry. Mummy berry disease affects both wild and cultivated blueberries and is found everywhere that blueberries are commercially grown (Gosch 2003; Schilder and Miles 2008; Florence 2014). Mummy berry disease accounted for $66,462 (8.1%) in highbush blueberry losses in British Columbia in 1969 (Pepin and Toms 1969). Infection rates as high as 85% have been reported in New Hampshire, and losses in organic production fields commonly reach nearly 100% (Wallace *et al.* 1976; Florence 2014). In 2002, in non-fungicide managed fields of rabbiteye blueberry in NC, a 70–80% crop loss was reported (Florence 2014). The fungus operates through two separate blighting phases of the disease, in which the first phase results in infection of young shoots, leaves, and emerging floral buds during favorable environmental conditions. The colonization of ovarian tissues characterizes the second blighting phase through the pollen tube pathway and subsequent necrosis of developing fruits (Shinners and Olson 1996; Ngugi and Scherm 2004, 2006; Tarnowski and Scherm 2005). *Mvc* utilizes wind, rain, and pollinating insects for spore dispersal to neighboring plants (Cox and Scherm 2001; McArt *et al.* 2016). Current methods for managing the disease include cultural practices and repeated fungicide use, but consistent control has been challenging to achieve (Schilder 2015; Dininny 2016).

Functional annotation studies provide a genetic basis for understanding the role(s) that fungal genes and proteins play in development, signaling, mating, host–pathogen interaction, and virulence (Amselem *et al.* 2011). However, these studies are heavily reliant on the accuracy of predicted gene models and the quality of the reference genome they are derived from. Draft genomes were recently developed and functionally characterized for three close relatives of *Mvc*: *Monilinia fructicola, Monilinia fructigena*, and *Monilinia laxa*, the causal pathogens of brown rot in pome and stone fruits (Abate *et al.* 2018; Rivera *et al.* 2018). These studies were able to provide insight into the process of brown rot disease induction and mating systems in other important species of *Monilinia*. It has been previously shown that *Mvc* displays a hemibiotrophic-like lifestyle with a mixed mating system and undergoes sexual outcrossing and inbreeding, which may create or maintain advantageous combinations of alleles (Burchhardt and Cubeta 2014, 2015; Burchhardt *et al.* 2017). These studies highlight the importance of understanding the mating system of *Mvc* and, in turn, how its mating system can contribute to genetic diversity and adaptation. Despite the importance of mummy berry disease in the blueberry industry, there is currently no reference genome for *Mvc* available, which could be used to study the infection strategy and mating system of the pathogen at a molecular level.

Pathogens must evade, withstand, or overcome host defense responses to induce disease (Kazan and Lyons 2014). They do so through the employment of secreted hydrolytic enzymes and effector proteins that act to degrade various substances, prevent pathogen detection, alter host defense responses, and change the host cellular environment to accommodate pathogen growth (Lo Presti *et al.* 2015). In this study, we show that *Mvc* contains a variety of putative secreted effector molecules for suppressing host defense responses and modulating plant host physiology. These effectors may function in both blighting phases of the disease. However, they would be considered particularly important to aid in the biotrophic phase, including during invasion of the ovary followed by fungal growth and development within blueberry fruit locules. Here, we also consider CAZymes and secondary metabolite (SM) biosynthesis enzymes as part of the genetic repertoire of fungal effectors since they or their products may also interact with host plant substrates.

Despite the economic importance of *Mvc*, to the best of our knowledge, there are no publicly available whole-genome sequences or functional gene data that can be used to understand important processes that occur throughout the *Mvc* life cycle. Very little or no molecular information about how *Mvc* interacts with its host, other competing fungi, and potential mates has been reported. However, as blueberries continue to grow in popularity and occupy more acreage, there is an increased benefit to obtaining high-quality reference genomes for pathogens such as *Mvc*. Reference genomes can be used to study the genetic basis of fungal development, mating systems, host specialization, and virulence (Perez-Nadales *et al.* 2014; De Miccolis Angelini *et al.* 2018; Xu *et al.* 2018). Understanding the genetic mechanisms underlying biological processes within *Mvc* provides a baseline and fundamental knowledge for developing and deploying sustainable disease-management solutions that target these specific processes.

Here, we report on (1) the use of PacBio long-read sequencing combined with Hi-C chromosomal contact data to develop the first comprehensive and publicly available genome assembly for the blueberry blighting fungus, *Mvc* isolate RL1, (2) the functional characterization of the *Mvc* genome using predicted gene models annotated by gene ontology (GO) terms, and (3) specific processes that contribute to the disease-causing ability of *Mvc*.

## Materials and methods

The *Mvc* isolate ‘RL1’ (*Mvc*-RL1) was previously described (Burchhardt and Cubeta 2014) and isolated as a single conidium from infected shoots of Southern Highbush Blueberry cv. ‘Legacy’ [Elizabeth × US75 (*V. darrowi*, ‘Florida 4B’, × ‘Bluecrop’)] in Ivanhoe, NC, USA.

PacBio RSII sequencing was performed at the University of Florida Interdisciplinary Center for Biotechnology Research (UF-ICBR). Hyphae of *Mvc*-RL1 was grown on a cellophane overlay of 1/2X PDA for 6 days, harvested by scraping the mycelia from the cellophane, and used to isolate high molecular weight (HMW) DNA with a CTAB method (Saghai-Maroof *et al.* 1984). DNA quality was evaluated using the Agilent TapeStation with a genomic tape. Quantitation was performed by fluorescence (Qubit, ThermoFisher). The HMW gDNA was applied to a G-tube (Covaris, Inc.) using fragmentation conditions for 20 kb. Large-insert (20 kb) library construction was performed according to the PacBio protocol (PN 100-938-400-02) with a few modifications. The final UFL library was used for sequencing with the PacBio RS II on two SMRT (Single-Molecule Real-Time) cells.

PacBio Sequel sequencing was performed at the North Carolina State University Genomic Sciences Laboratory (NCSU-GSL). The *Mvc*-RL1 mycelial plugs were recovered from storage (−80°C) by plating on Petri dishes containing 1/2X potato dextrose agar (PDA, Difco). Expanding mycelia were subsequently transferred to flasks of 1/2X potato dextrose broth (PDB, Difco). The mycelia in 1/2X PDB were vacuum filtered and stored at −20°C. The HMW gDNA was extracted from the mycelia using a CTAB method (Amir *et al.* 2015), and the quantity and quality were determined with Qubit and pulsed-field gel electrophoresis (PFGE), respectively. DNA was separated by size fractionation with the SageELF system (Sage Sciences, Beverly, MA, USA) and a target size of 10 kb. Large-insert (10-20 kb) libraries were prepared according to the PacBio protocol (PN: 100-938-400-03) with size-selection steps omitted, as the input gDNA was previously size-selected using the SageELF system and of an appropriate size for library construction. The sequencing of the final NCSU library was performed with the PacBio Sequel on four SMRT cells.

Genomic Hi-C libraries were prepared by Phase Genomics (Seattle, WA, USA) with the Proximo Hi-C Kit from 0.2 g of *Mvc*-RL1 mycelial tissue grown in 1/2X PDB and sequenced at NCSU-GSL on the Illumina NextSeq 500 using 2 × 150 bp PE chemistry.

Multiple draft assemblies were generated using two different assembly programs, HGAP4 (SMRTLink v4.0) (Chin *et al.* 2013) and Canu (v1.6) (Koren *et al.* 2017), with different parameters and combinations of RSII and Sequel data (File S1). The two highest quality genome assemblies were aligned using the Nucmer module of Mummer (Marçais *et al.* 2018) and chosen for further comparison based on the longest contig N50 and lowest number of contigs. RepeatModeler v1.0.8 (Smit and Hubley 2015) was used to generate *de novo* repetitive element predictions for the *Mvc* genome, which was used by RepeatMasker v4.0.6 (Smit *et al.* 2015) as the search library for masking and to generate a summary table of identified repetitive elements. Gene models of two assemblies were predicted using Maker (v2.31.8) (Campbell *et al.* 2014) with two cycles of the pipeline using the reviewed proteins in Uniprot database as the protein evidence and RNA-Seq (Illumina Hiseq) transcripts as the EST evidence. In the first cycle of the Maker program, only GeneMark-ES v4.32 (Ter-Hovhannisyan *et al.* 2008) was used. Gene models predicted by the Maker with a strong annotation quality score (AED = 0) were used for Augustus v3.2.2 (Stanke *et al.* 2006) training. In the second cycle of Maker, Augustus, and GeneMark were used for gene prediction. A custom repeat library was generated by combining Dfam_3.0 (Hubley *et al.* 2016), RepBase-20181026 databases (Bao *et al.* 2015), and a de novo species-specific library constructed using RepeatModeler v2.0.1 (Flynn *et al.* 2020). RepeatMasker v4.0.6 (www.repeatmasker.org/) was used to mask repeats in the genome assembly upon the custom library. BUSCO v1.22 (Benchmarking Universal Single-Copy Orthologs) (Simão *et al.* 2015; Waterhouse *et al.* 2017) was run on predicted gene sets of the two assemblies to assess the completeness of each assembly. The genome assembly with the longest contig N50, most discernable nuclear contigs, and highest BUSCO score was the HGAP4-derived assembly. Sequences of individual repeat families were aligned with ClustalW v2.1 (Larkin *et al.* 2007) and subjected to an alignment-based analysis with RIPCAL v2.0 (Hane and Oliver 2008) using the degenerate consensus model to identify dominant forms of Repeat-Induced Point mutations (RIP), in which C and G nucleotides are converted to T and A nucleotides, respectively. The degenerate consensus model was chosen for RIPCAL alignment-based analysis of the *Mvc* genomic repeats due to the variable lengths of aligned sequences. Scaffolding by Phase Genomics was performed on only the nuclear contigs of the HGAP4 assembly using Hi-C data. Mis-assembled and mis-oriented contigs in the genome were manually corrected in Juicebox v1.8.8 (Rao *et al.* 2014; Durand *et al.* 2016; Dudchenko *et al.* 2017, 2018). Approximate positions of centromeres were determined by zooming in on the Hi-C heatmap in Juicebox and selecting the region corresponding to a centromeric signature (Figure 2). The centromeric regions were analyzed for tandem repeat content using Tandem Repeat Finder (TRF) with default settings and globally aligned using a BLAST all-vs-all technique. The *Mvc* genome was analyzed for AT-rich regions with OcculterCut v1.1 (Testa *et al.* 2016). To identify genomic regions potentially affected by RIP mutation, RIP-index scan analysis with RIPCAL v2.0 was run on the *Mvc* scaffolds with default settings to identify regions with a high RIP index.

Gene models were imported into Omicsbox v1.2 for functional annotation using the NCBI NR (Non-Redundant) protein, EggNOG (evolutionary genealogy of genes: Non-supervised Orthologous Groups) Mapper 5.0, and KEGG (Kyoto Encyclopedia of Genes and Genomes) databases. InterProScan was used for annotating sequences based on protein domains and families (Huerta-Cepas *et al.* 2017, 2019).

InterProScan and eggNOG annotations were merged with BLAST-based annotation results to build a comprehensive set of functional GO terms for the predicted *Mvc* proteome. To test for enrichment of GO terms, sequence subsets were compared against the entire set of predicted gene models using a Fisher’s Exact Test with an FDR-corrected p-value of 0.01. Secondary Metabolite Unique Regions Finder (SMURF, http://smurf.jcvi.org/index.php), a web-based software, was used to identify SM biosynthesis genes based on genomic clustering and protein domain content (Khaldi *et al.* 2010). Signal peptides were identified in the Maker dataset by using SignalP v5.0 prediction software (Petersen *et al.* 2011; Almagro Armenteros *et al.* 2019b, 2019a). Putative effectors for *Mvc* were identified by running EffectorP v2.0 (Sperschneider *et al.* 2016, 2018) on the genes with signal peptides that were identified by SignalP. Putative effector candidate genes identified by EffectorP were mapped to the nine *Mvc* genomic scaffolds utilizing ggplot2 package available in Rstudio (Wickham 2016). dbCAN2 was used for predicting putative CAZymes in the *Mvc* gene models (Zhang *et al.* 2018).

### Data availability

The genome sequences of *Mvc, and raw PacBio Read* are accessible in GenBank under BioProject number PRJNA479660, Genome Files CP063405-CP063413, and SRA: SRR11278194. The *MAT* locus sequences for the reference genome isolate RL1, and the deletion allele from isolate NCWL-11 are available through GenBank Accession MW176035.

Supplementary material is available at figshare DOI: https://doi.org/10.25387/g3.12276584.

## Results

### Genome assembly and quality assessment

Sequencing results are presented in Supplementary Tables S1 and S2. A total of 12.4 Gb of raw data (413X genome coverage) was generated with PacBio RSII and Sequel systems. RSII generated longer sequence reads than the Sequel system due to differences between genomic libraries and optimization conditions. The two libraries were constructed independently at UF and NCSU. A total of 26 Gb of raw Hi-C data (866X genome coverage) was generated for genome scaffolding.

Two assemblies with the highest quality based on six iterations using HGAP4 and Canu were developed using combined Sequel and RSII data. The HGAP4 assembly had 59 polished contigs with an N50 length of 3.6 Mb, and a total length of 31 Mb, whereas the Canu assembly had 20 polished contigs, with an N50 of 2.4 Mb, a total length of 30.7 Mb. Contig sizes and makeup for the HGAP4 assembly are summarized in Supplementary Table S3. The two assemblies were annotated with the Maker pipeline and predicted gene sets evaluated using BUSCO. The HGAP4 and Canu assemblies produced BUSCO scores of 1423 and 1418, respectively (Table 1).

**Table 1.** BUSCO analysis results for *Monilinia vaccinii-corymbosi* assemblies before and after scaffolding with Hi-C data

Analysis metric	Canu_20 assembly	**%** ^*^	HGAP4_59 assembly	**%** ^*^	HGAP4_59-HiC assembly	**%** ^*^
Complete	1,418	98.6	1,423	99.0	1,410	98.1
Complete and single	1,252	87.1	1,263	87.8	1,257	87.4
Complete and duplicated	166	11.5	160	11.1	153	10.6
Fragment	15	1.0	11	0.8	23	1.6
Missing	5	0.3	4	0.3	5	0.3

* Of a total of 1,438 conserved genes.

**Figure 1.**
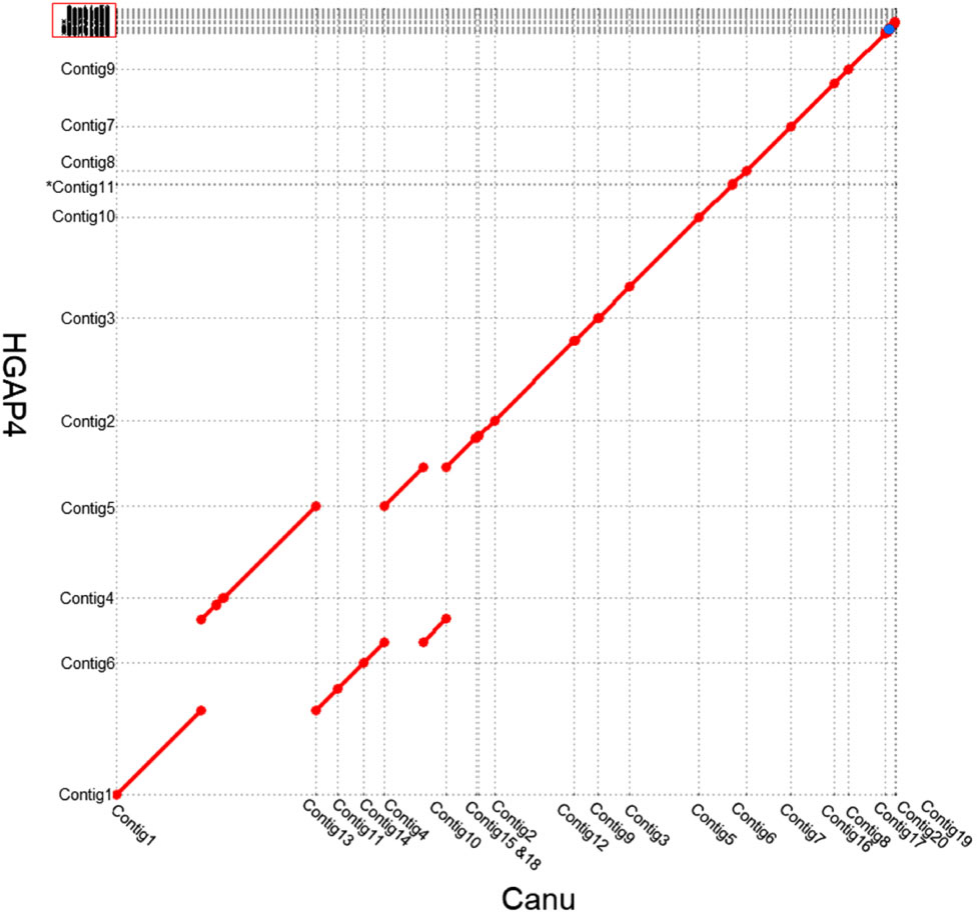
Global alignment of the two *Monilinia vaccinii-corymbosi* assemblies. Canu assembly (20 contigs, *x*-axis) and HGAP4 assembly (59 contigs, *y*-axis). Contigs from the HGAP4 assembly mapping to mitochondrial and rDNA contigs in the Canu assembly (contigs 17, 19 and 20) are marked with a red box. Asterisk (*) denotes HGAP4 contig 44 (10.69 kb) encoding a retrotransposon and low complexity sequences (not labeled).

**Figure 2.**
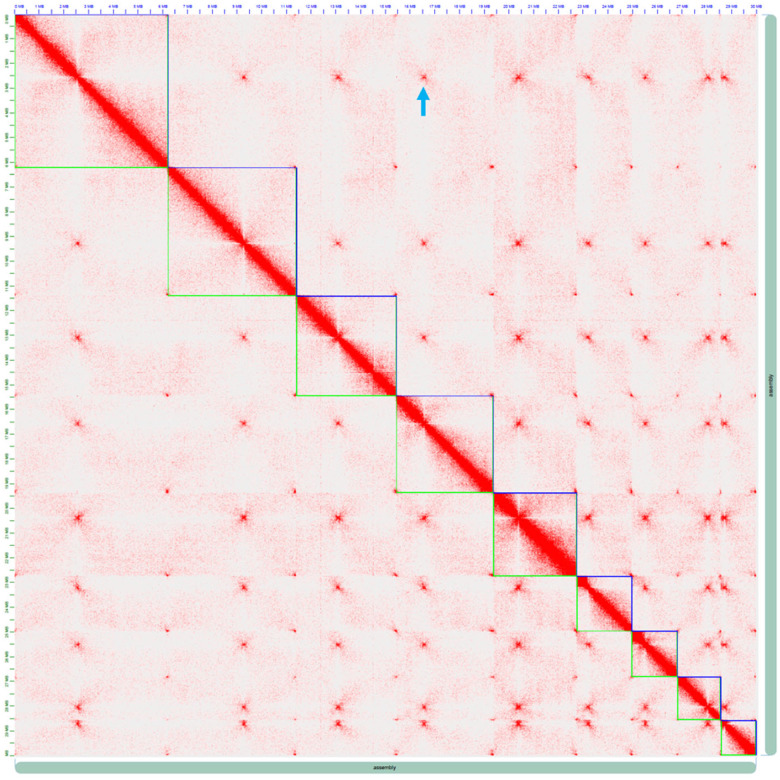
Heatmap visualization of the *Monilinia vaccinii-corymbosi* genomic scaffolds. Red indicates regions of genomic interaction. The blue arrow shows a representative centromeric interaction point.

Dot plot comparisons showed high levels of collinearity and synteny between the two assemblies (Figure 1). The largest contig (7.8 Mb) in the Canu assembly had synteny with three contigs in HGAP4 contigs and may have been merged to form this large contig. Twelve contigs from the HGAP4 assembly mapped to 17 contigs from the Canu assembly determined by Nucmer analyses and represented the nuclear genome. However, one of the nuclear contigs was small (10.69 kb) and encoded a retrotransposon and low complexity sequences. Of the 59 HGAP4 contigs, 39 were determined by Blastn and Blastx analyses to represent components of the mitochondrial genome (contigs 17 and 20 in the Canu assembly), 8 contained rDNA repeats (contig 19 in the Canu assembly), and 12 represented the nuclear genome. The HGAP4 assembly represents the nuclear genome of *Mvc* on 12 contigs totaling 30.2 Mb, and the Canu assembly represents the nuclear genome on 18 contigs totaling 30.3 Mb. Dot plots generated with contigs from both *Mvc* assemblies, and the *Botrytis cinerea* and *S. sclerotiorum* reference genomes (GenBank assembly accessions: GCA_000143535.4 and GCA_001857865.1, respectively) indicated greater collinearity between the *Mvc* HGAP4 assembly and these complete genome sequences (data not shown). Based on BUSCO scores and assembly metrics (larger contig N50, fewer nuclear contigs, and greater collinearity with reference genomes of closely related species), the HGAP4 assembly with 59 contigs was chosen as the highest quality assembly of the *Mvc* nuclear genome, and the 11 large nuclear contigs were utilized for scaffolding with Hi-C data. The final *Mvc* RL1 genome comprises nine chromosomes with a scaffold N50 length of 4 Mb and spans a total length of 30 Mb (Figure 2; Tables 2 and 3). The repeat content of the assembled genome was estimated to be 5.98% of the total genome sequence content. The largest fractions included simple repeats (2.46% of the genome) followed by interspersed repeats (2.35% of the genome). The majority of these interspersed repeats were unclassified (1.65% of the genome), but a significant portion (0.67% of the genome) were members of the TY1/*Copia* and TY3/*Gypsy* families of long terminal repeats (LTR retroelements) (Table 4).

**Table 2.** Statistics for individual chromosome-scale scaffolds of the *Monilinia vaccinii-corymbosi* (*Mvc*) strain RL1 genome

Feature	Length (bp)	Number of gaps	Total gap length (bp)
Mvc-RL1_Chromosome1	6,208,549	1	100
Mvc-RL1_Chromosome2	5,195,418	0	0
Mvc-RL1_Chromosome3	4,062,641	0	0
Mvc-RL1_Chromosome4	3,929,668	0	0
Mvc-RL1_Chromosome5	3,356,619	0	0
Mvc-RL1_Chromosome6	2,240,868	0	0
Mvc-RL1_Chromosome7	1,853,551	1	100
Mvc-RL1_Chromosome8	1,745,410	0	0
Mvc-RL1_Chromosome9	1,415,376	0	0
Total	30,008,100	2	200

**Table 3.** Genome statistics for Hi-C scaffolded *Monilinia vaccinii-corymbosi* (*Mvc*) strain RL1 genome

Feature	*Mvc* genome
Size (bp)	30,008,100
% GC	44.53
% Repeat	3.46
Total gap length (bp)	200
Number of scaffolds	9
Longest scaffold (bp)	6,208,549
Shortest scaffold (bp)	1,415,376
Scaffold N50 length (bp)	4,062,641
Number of contigs	59
Longest contig (bp)	5,195,418
Shortest contig (bp)	540,959
Contig N50 length (bp)	3,635,407

**Table 4.** Repetitive elements identified within the *Monilinia vaccinii-corymbosi* (*Mvc*) assembly

	Number of elements	Total length (bp)	Percent of total repetitive elements	Percent of the assembled genome
Total interspersed repeats	2,636	1,151,796	57.60	3.84
Retroelements	0	0	0.00	0.00
SINEs	0	0	0.00	0.00
LINEs	81	151,996	7.60	0.51
LTR elements	416	467,492	23.38	1.56
DNA transposons	69	18,586	0.93	0.06
Unclassified	2,070	513,722	25.69	1.71
Small RNA	0	0	0.00	0.00
Satellites	0	0	0.00	0.00
Simple repeats	19,013	741,424	37.08	2.47
Low complexity	2,110	107,011	5.35	0.36

Similar to other filamentous fungi, the centromeres of *Mvc* range from ∼29 to 41 kb (average of 34.1 kb) in length, as estimated by using the Hi-C heatmap. Based on estimated lengths of centromeric regions and analyses for GC content, gene number, and repeat content, the only nuclear scaffold that did not appear to have a true centromere was Mvc_Scaffold_07 (refer to Supplementary Table S4); therefore, this scaffold is excluded from the following results about the *Mvc* centromeres. Although the overall GC content of the *Mvc* genome is 44.4%, the *Mvc* centromeres range from 17.7% to 33.8%, indicating AT-richness and possible RIP (Smith *et al.* 2012). The *Mvc* centromeres are also depleted of transcriptional activity and contain only 3–6 genes per region. However, tandem repeats are abundant within the *Mvc* centromeres, and tandem repeat analysis revealed an occurrence of 32–106 tandem repeats per centromere. A global alignment of centromeric region sequences resulted in an average of 87.2% similarity between them, with the longest alignment length being 6.7 kb.

AT-rich region analysis with Occultercut v1.1 split the *Mvc* genome into two separate regions based on GC content. Region 0 (R0) had GC content of 0–34.4%, with an average peak at 30.1% and encompassed 2.9% of the entire genome. The portion of the genome contained in this AT-rich region also only contained 66 total genes, indicating that this portion of the genome is less transcriptionally active. Based on the AT-richness and lack of genes in R0, it is likely that this region contains primarily centromeres and RIP-affected sequences. Region 1 (R1) had GC content of 34.4–100%, with an average peak at 45.1% and encompassed 97.1% of the entire genome. The distribution of GC content across the entire *Mvc* genome is presented in Supplementary Figure S1. RIP-index scan of the *Mvc* genome indicated that 16,346 regions have been affected by RIP mutations. The average length of the RIP-affected regions is 534 bp, and the distribution of regions across the *Mvc* genomic scaffolds can be seen in Supplementary Figure S2. Alignment-based RIP analysis of the individual repeat families identified in the *Mvc* genome revealed that CT to TT di-nucleotide mutation was the dominant form of RIP (Supplementary Table S5).

### Genome annotation

Gene prediction using the Maker pipeline identified 9,399 gene models in the scaffolded *Mvc* genome. Summary statistics of the Maker gene models are presented in Table 5. By using three strategies, BLAST, InterProScan, and EggNOG mapper, a comprehensive set of GO terms from homology-based, protein domain- and family-based, and phylogeny-filtered sources was generated and assigned to the predicted gene models. A total of 94% of *Mvc* gene models had significant BLAST hits. InterProScan and EggNOG Mapper increased the percentage of *Mvc* gene models with functional annotation. The percentage of annotated sequences rose from 64% to 68% to 72% with BLAST, InterProScan, and EggNOG Mapper, respectively. Approximately 6% of the sequences (573) could not be functionally annotated with at least 1 of the database sources used. The unannotated sequences were >3.5× shorter in average length than the overall set of gene models [*e.g.*, 139 *vs* 491 amino acids (aa), respectively] (Supplementary Figure S3). The top-hits by species distribution showed the highest similarities with *M. fructicola*, *M. laxa*, and *Sclerotinia borealis*.

**Table 5.** Statistics for *Monilinia vaccinii-corymbosi* (*Mvc*) gene models predicted by Maker

Metric	*Mvc* gene models
Predicted protein-coding genes	9,399
Gene density (# of genes per Mb)	313.3
Average protein length (aa)	492.8
Average transcript length (bp)	1,489
Total transcript length (bp)	13,992,245
Transcript coverage of the genome	46.6%
Exons per gene (average)	2.9
Average exon length (bp)	511.3

**Figure 3.**
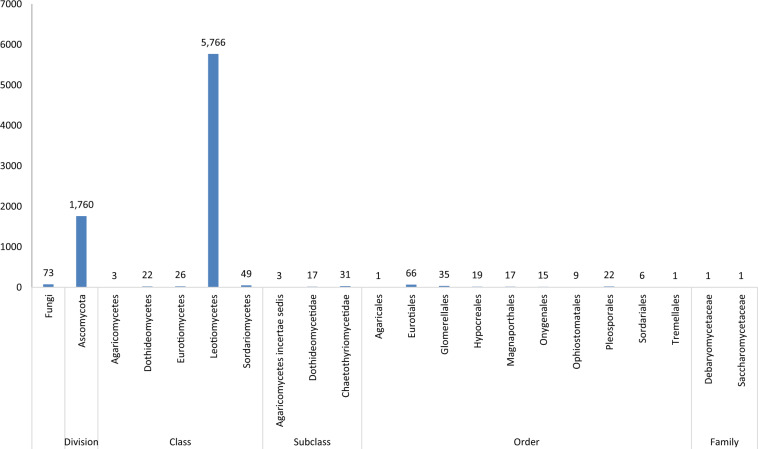
Orthologous analysis results of *Mvc* predicted gene sequences using eggNOG Mapper 5.0.

Orthologous analysis with EggNOG identified a total of 8,201 orthologs in the *Mvc* predicted genes, and 7,943 of these were from fungi. Most of the orthologs were conserved among members of the Leotiomycetes, the class in which *Mvc* resides (Figure 3). Orthologous genes important in pathogenicity were identified, including those encoding pectinolytic enzymes (MvcIVH1_00092) and proteins that promote invasive growth (MvcIVH1_00579) and SM production (MvcIVH1_05756).

### Putative effectors

**Figure 4.**
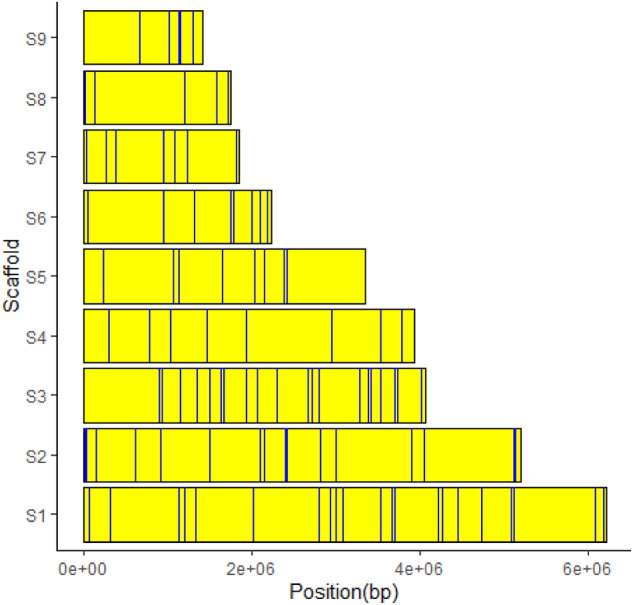
Effector gene positions in each of the 9 scaffolds of the *Monilinia vaccinii-corymbosi* genome assembly.

EffectorP 2.0 predicted 105 effectors from 774 sequences containing signal peptides (1.1% of the total predicted *Mvc* genes). Some of the putative effectors found by EffectorP in *Mvc* include a necrosis- and ethylene-inducing protein 1 precursor, chorismate mutase, and hydrolytic enzymes. Thirty-five (33%) of the predicted effectors had no BLAST hit, and can be considered putative novel effectors in *Mvc*. The predicted effector sequences had BLAST top-hits for *M. fructicola* (20 hits), followed by *M. laxa* (11 hits) and *S. sclerotiorum* (7 hits). Other flower-infecting fungi, such as *Botryotinia convoluta* and *Botrytis elliptica* had 4 BLAST top-hits. Direct GO term counts revealed that the putative effectors are involved in processes characteristic of the fungal secretion system, including protein localization and transport (Supplementary Figure S4). Relative to the entire predicted *Mvc* proteome, the 105 putative effector sequences were not enriched for any GO terms. However, the enrichment results for GO cellular components displayed that they are depleted in intracellular organelles and the cytoplasm (Supplementary Table S6), providing additional evidence that they are likely to be in the secretion pathway. Based on direct GO term counts and GO term enrichment results, we can deduce that the putative effectors identified by EffectorP are secreted to the extracellular environment and may play important roles in manipulating host defense and establishing disease compatibility. The putative effector candidate genes were distributed across all nine scaffolds and did not show a strong propensity for sub-telomeric localization (Figure 4). Effector gene clustering was observed, particularly on scaffold 2 and on scaffolds 8 and 9.

### CAZymes

In *Mvc*, 366 sequences were predicted to be CAZymes by ≥2 dbCAN2 databases (HMMER, DIAMOND, and Hotpep), which represent 375 total CAZymes in 111 families. Glycoside hydrolases (GH) was the largest CAZy module with 198 proteins, followed by glycosyltransferases (GT) with 89, auxiliary activities (AA) with 48, carbohydrate esterases (CE) with 21, carbohydrate-binding modules (CBM) with 14, and polysaccharide lyases (PL) with 5. *Mvc* contains 327 CAZymes in the CBM+CE+GH+GT+PL modules. The distribution of predicted CAZymes in *Mvc* is presented in Supplementary Table S7. Pathogenesis (11 sequences), pectin catabolism (15 sequences), and oxidation-reduction (35 sequences) were among the top biological processes inferred from direct GO term counts for *Mvc* CAZyme sequences (Supplementary Figure S5). *Mvc* CAZyme sequences are enriched for GO biological processes involved in the degradation of plant-derived carbohydrates and the development of new fungal cells, including cellulose and pectin catabolism, carbohydrate metabolism, fungal cell wall biogenesis, and biofilm formation (Supplementary Table S8).

### Secondary metabolites

In an analysis of secondary metabolism potential, SMURF identified 90 SM biosynthesis-related genes in 15 clusters across the *Mvc* genome. Each cluster contained at least one backbone core synthase gene encoding a non-ribosomal polyketide synthase (NRPS), NRPS-like, polyketide synthase (PKS), PKS-like, or hybrid enzyme (NRPS-PKS hybrid) gene and several “supporting genes,” including transporters, transcription factors, and tailoring enzymes. Twenty-three genes encoded the core SM synthase genes, 16 of which were within one of the 15 clusters, and 7 of which were identified but not assigned to a cluster (Supplementary Tables S9 and S10). Each *Mvc* chromosome contained at least one SM cluster except chromosome 5. Chromosome 1 contained four SM clusters. SM biosynthesis clustering results are presented in Table 6, and the full list of *Mvc* genes identified as SM biosynthesis genes are presented in Supplementary Table S11. One SM cluster identified contained putative HC-toxin synthetase (a host-selective toxin than inhibits histone deacetylases) and the MFS transporter protein-encoding gene. As such, the Mvc protein sequence showed 83% aa similarity to a putative HC-toxin synthetase protein from *B. cinerea*. This cluster also contains a putative pyrroline-5-carboxylate reductase protein and an alcohol dehydrogenase. Pyrroline-5-carboxylate reductase (Pro3) plays a role in L-proline biosynthesis, a precursor for synthesizing HC-toxin. A direct GO term count revealed the SM biosynthesis-related genes identified by SMURF play active roles in oxidation-reduction, transmembrane transport, pathogenesis, transcriptional regulation, and biosynthesis processes (Supplementary Figure S6). An enrichment test for GO terms in the SMURF-predicted SM biosynthesis genes showed they were enriched for the oxidation-reduction process, pathogenesis, and interspecies interaction between organisms (Supplementary Table S12).

**Table 6.** Number of secondary metabolite gene families predicted by SMURF in *Monilinia vaccinii-corymbosi*

Secondary metabolite	Genes
PKS	10
PKS-like	2
NRPS	5
NRPS-like	5
PKS-NRPS hybrid	1
DMATS	0
Total	23

### Other secreted proteins

The *Mvc* genome encodes 774 proteins with a secretory signal peptide predicted by SignalP, representing 8.23% of the total predicted proteins. Of the identified secreted proteins, 53 (6.85%) and 6 (0.78%) show predicted peptidase and lipase activity, respectively. Signal peptides predicted by SignalP were expected to overlap with disease-related proteins identified by other prediction software tools, such as SMs, effectors, and CAZymes, which are secreted by fungi during infection.

Common predicted proteins include 185 CAZymes predicted by dbCAN2, 105 putative effectors predicted by EffectorP, and 5 SM biosynthesis pathway proteins predicted by SMURF. Direct GO counts and enrichment tests functionally confirm that these 774 proteins are part of the *Mvc* secretome based on their molecular activity and cellular location. These proteins are primarily active in the extracellular region, cell wall, cell membrane, and intracellular organelles in the secretory pathway (ER, Golgi, vacuole, and vesicle transport) (Supplementary Figure S7). Predicted molecular functions include hydrolase activity, metal ion binding, oxidoreductase activity, cellulose-binding, and polygalacturonase activity (Supplementary Figure S8). Compared to the entire set of *Mvc* predicted genes, sequences containing signal peptides identified by SignalP are enriched for biological processes related to cleavage and utilization of plant cell wall components and fungal stress response (Supplementary Table S13). Among the signal peptide sequences, pectin catabolism through polygalacturonase activity, proteolysis, fungal cell wall biogenesis, and drug catabolism are some of the processes *Mvc* appears to utilize to access nutrients from the plant host while simultaneously protecting itself from environmental stressors. These results indicate the importance of *Mvc* secreted proteins in host–pathogen interaction, fungal cell maintenance during infection, and disease induction.

### Mating in *Monilina vaccinii-corymbosi*

**Figure 5:**
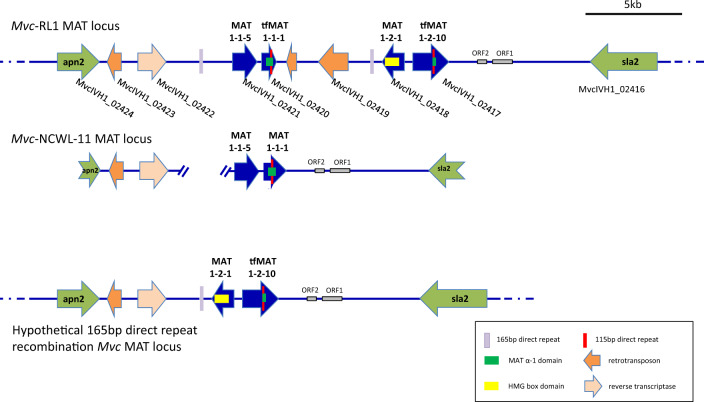
Structure of the *Monilinia vaccinii-corymbosi MAT* locus of reference genome isolating RL1 (*Mvc*-RL1).

Mating-type determinants common to Ascomycota fungi were identified in *Mvc*-RL1, and the *MAT* locus structure was characterized. The *Mvc MAT* locus was located on chromosome 3 and spanned 25.5 kb. Within the locus, sequences of four *MAT* genes, *MAT1-1-5*, tf*MAT1-1-1*, *MAT1-2-1*, and tf*MAT1-2-10* were identified in this order flanked by the *APN2* (putative DNA lyase) and the *SLA2* (cytoskeleton assembly control) genes. The structure of the *Mvc*-RL1 *MAT* locus is shown in Figure 5 and Tables 7 and 8, and Supplementary S14. Other sequences were identified within the *Mvc MAT* locus, including three retrotransposons, two open reading frames (ORFs), and one reverse transcriptase. One of the retrotransposons and both ORFs (ORF1 and ORF2) were not called as genes during gene prediction. The sequence of ORF1 (960 bp) encodes or is a pseudogene of a partial reverse transcriptase. ORF2 (476 bp) encodes an unknown sequence with no significant BLAST hits. A substantial number of Illumina reads aligned to both of the ORFs, indicating that they are transcriptionally active. All four mating determinant genes were also transcriptionally active, although relatively few transcripts mapped to *tfMAT1-1-1* (384 ± 115) and *tfMAT1-2-10* (729 ± 86) relative to *MAT1-2-1* (2461 ± 131) and *MAT1-1-5* (3943 ± 130) grown in culture.

**Table 7.** Statistics for *Monilinia vaccinii-corymbosi* mating-type locus (*MAT*) genes

*Mvc*-RL1 *MAT* locus gene	Direction	Gene length (bp)	Number of introns	Number of exons	CDS length (bp)	Protein length (aa)
*APN2*	–	2,169	4	5	1,929	642
retrotransposon	+	711	0	1	711	236
reverse transcriptase	–	1,446	2	3	528	175
*MAT1-1-5*	–	1,038	2	3	913	289
*tfMAT1-1-1* ^*^	–	727	1	2	678	225
unknown	+	1,593	3	4	1,437	478
*MAT1-2-1*	+	1,172	2	3	1,065	354
*tfMAT1-2-10* ^*^	–	1,817	4	5	1,263	420
*SLA2*	+	3,499	4	5	3,165	1,054

* Truncated and fused *MAT* genes.

**Table 8:** Statistics for intergenic regions within the *Monilinia vaccinii-corymbosi* mating-type locus (*MAT*)

Intergenic region	Length (bp)
*APN2*—MAT1-1-5^*^	6,901
*MAT1-1-5—tfMAT1-1-1*	497
*tfMAT1-1-1—MAT1-2-1* ^*^	5,513
*MAT1-2-1—tfMAT1-2-10*	507
*tfMAT1-2-10—SLA2*	6,909

* Region contains predicted coding sequences of non-*MAT* genes.

The *Mvc MAT1-1-5* and *MAT1-2-1* genes share strong sequence homology, intron-exon structure, and encoded proteins with corresponding orthologs from *M. laxa*, *M. fructicola*, *M. fructigena*, and other Sclerotiniaceae species. The high-mobility group (HMG) box domain (IPR009071) present in MAT1-2-1 is encoded by nucleotides 612-875 bp downstream of the ATG start codon and is interrupted by one intron.

The *Mvc tfMAT1-1-1* gene is 727 bp (225 aa) in length with one intron and appears to be truncated and fused with other gene sequences. We designated this gene *tfMAT1-1-1* to indicate the truncated and fused nature of this sequence. The coding sequence encodes a 225 aa peptide containing a nearly complete MAT alpha 1 domain (IPR006856). The length of the *Mvc tfMAT1-1-1* gene is shorter than the lengths reported for most of the closely related homologs. Closer examination of the sequence revealed that homology with other MAT1-1-1 proteins ends in the C-terminus of the tfMAT1-1-1 protein ends three codons downstream of a 115 bp direct repeat sequence found within the IPR006856 alpha 1 domain. The final 21 codons encode 20 amino acids with strong sequence homology with APN2 proteins from other Sclerotiniaceae spp., indicating a potential gene fusion may have occurred as a result of recombination within the *MAT* locus.

A second identical copy of the 115 bp direct repeat is present in the *Mvc tfMAT1-2-10* gene. This gene is 1,817 bp (420 aa) in length with four introns. The coding sequences encode a 420 aa peptide. At nearly double the size of the *MAT1-2-10* genes from *M. laxa*, *M. fructicola*, and *M. fructigena* (957 bp), this is the longest length reported for a *MAT1-2-10* gene among all close relatives examined. Examination of the C-terminus of the tfMAT1-2-10 protein (∼1/3 of the entire peptide) revealed strong homology to the C-terminus of MAT1-1-1 proteins from other Sclerotiniaceous fungi. The 115 bp direct repeat motif in the tf*MAT1-2-10* sequence is largely found within intron number three. The final 15 bp of this direct repeat motif initiates the 5′ end of exon 4. These sequences and the sequences encoding the 146 amino acids downstream of them share very high sequence similarity with MAT1-1-1 proteins from other Sclerotiniaceae spp.

Direct and indirect repeat sequences within the *MAT* loci of *Sclerotinia* species have been reported to facilitate intra-locus recombination during meiosis resulting in the deletion or rearrangement of *MAT* genes (Chitrampalam *et al.* 2013; Xu *et al.* 2016). The 115 bp direct repeat sequences share significant sequence identity to the 250, 256, and 146 bp *MAT* locus repeats described from *Sclerotinia spp.* (Supplementary Figure S9). Recombination between the *Mvc* 115 bp direct repeats would result in the in-frame restoration of the *MAT1-1-1* coding sequence, including the C-terminus of the alpha 1 domain and the deletion of intervening sequences including the *MAT1-2-1* and *MAT1-2-10* sequences as diagrammed in Figure 5. PCR screening of isolates from populations previously reported to show random mating (Burchhardt and Cubeta 2014) identified two isolates of seven screened with an amplicon consistent with an intra-locus recombination between the 115 bp direct repeat copies. The locus of one isolate, NCWL-11, was sequenced. The sequencing results confirmed that only a single copy of the 115 bp repeat was present, the complete *MAT1-1-1* coding sequence was restored in-frame, and a deletion of sequences present between the two 115 bp direct repeat sequences in the reference genome including the coding sequences of *MAT1-2-1* and the 5ʹ end of *tfMAT1-2-10* were deleted (Figure 5). In addition to the 115 bp direct repeat sequences found in the *Mvc tfMAT1-1-1* and *tfMAT1-2-10* genes, the intergenic regions were repeat-rich with numerous tandem, direct and inverted repeats, including a 165 bp near-identical (99%) direct repeat upstream of *MAT1-1-5* and downstream of *tfMAT1-1-1*. If these sequences were to recombine during meiosis, similar to what appears to have occurred between the 115 bp direct repeat in isolate NCWL-11, progeny lacking the *MAT1-1-5* and *tfMAT1-1-1* sequences would result. This hypothetical allele of the *Mvc MAT* locus is diagrammed in Figure 5. This arrangement of the *MAT* locus was also screened by PCR with the same seven isolates used above but not detected. The *MAT* locus sequences for the reference genome isolate RL1, and the deletion allele from isolate NCWL-11 are available through GenBank Accession MW176035.

Many additional genes potentially involved in vegetative and mating compatibility were identified in the *Mvc* genome. These include 11 genes annotated for mating-type switching processes. These genes had top BLAST hits from *M. laxa*. Fungi communicate mating compatibility through the use of pheromone signals. Fifty-four proteins that annotated for roles in pheromone signaling were identified in *Mvc*, 16 of which were within the GO mating subset. The process of vegetative cellular fusion is a form of somatic recognition that allows fungi to exchange genetic material either with themselves or individuals of another genotype without undergoing zygote formation or meiosis (Saupe *et al.* 2000; Dementhon *et al.* 2006; Read and Roca 2006; Choi *et al.* 2012). Several genes involved in cellular fusion (30) and heterokaryon incompatibility (20) were identified in *Mvc*. Heterokaryon incompatibility proteins prevent the fusion of hyphal cells between two genetic individuals with different alleles at *het* loci. A summary of these annotations is presented in Table 9, and the full list of *Mvc* genes is presented in Supplementary Table S15.

**Table 9:** Mating-related functions found within *Monilinia vaccinii-corymbosi* mating-type locus (*MAT*) maker functional annotations

Biological process	Number of sequences
Mating type switching (GO:0007533)	11*
Gene conversion at mating-type locus (GO:0007534)	10*
Pheromone signaling (GO:0090538; 0010969; 0036318; 0000772)	16*
Cellular fusion (GO:0000747)	30*
Heterokaryon incompatibility (InterPro: IPR010730; IPR010816)	20

Asterisks (*) denotes functions identified by gene ontology (GO) terms. Only parental GO terms are listed for processes that had multi-level GO hierarchies.

## Discussion

Agricultural losses due to mummy berry disease of blueberry contribute to reductions in fruit quantity and increased production costs. Despite the production and economic losses resulting from mummy berry disease, there was no reference genome or functional genomic data previously available for the causal pathogen *Monilinia vaccinii-corymbosi* (*Mvc*). Structural annotation of the assembly was used to characterize the *MAT* locus and repeat the content of the *Mvc* genome. Functional annotation was used to understand how *Mvc* utilizes effectors during fruit blighting of blueberry and to identify proteins contributing to genetic exchange through mating and cellular hyphal fusion.

During fruit blighting, conidia are deposited by pollinators onto the stigma of a compatible host flower. Subsequently, the *Mvc* hyphal germ tube is able to follow the pollen tube pathway down the style and into the ovary (Shinners and Olson 1996; Ngugi and Scherm 2004). The hyphal germ tube is believed to go undetected by the host, as no evidence of a hypersensitive response in infected blueberry styles has been documented. The ability for *Mvc* to avoid host-mediated detection during this process can likely be attributed to (1) fungal protein products that mimic plant pollen and (2) prevention and mitigation of eliciting host defense signals. Evidence from the predicted gene model annotation suggests that *Mvc* utilizes biotrophic effectors to alter host defenses during flower style infection and fruit locule colonization. Synthesis of salicylate is one of the mechanisms used by plants to defend themselves against pathogens. However, some fungal pathogens are able to degrade host produced salicylate or its precursors, including isochorismate (Penn and Daniel 2013; Liu *et al.* 2014). In this study, two transcripts encoding isochorismatase enzymes were identified in *Mvc*, indicating that the fungus may be able to prevent the accumulation of host salicylate through degradation of the precursor, isochorismate. The effectors identified in *Mvc* provide molecular evidence of an infection style involving necrosis of host tissue and degradation of host salicylic acid.

Fungal CAZymes and SMs play crucial roles in nutrient acquisition, host cell-wall degradation, and adaptation to stress. Pathogens must directly or indirectly penetrate the host plant cell wall to reach nutrient reservoirs inside the cell, and the GH, PL, and CE CAZyme modules are known to contain plant cell-wall degrading enzymes (PCWDEs) (Lopez *et al.* 2018). The functional annotation using GO terms of the 366 predicted CAZymes in *Mvc* elucidates that these CAZymes play an important role in host tissue degradation and colonization of the fruit pericarp. As a result of CAZyme activity, *Mvc* can necrotize young shoots and floral buds during the first blighting stage and colonize, then necrotize fruit in the second blighting stage of mummy berry disease.

Initially identified as a host-selective toxin in *Cochliobolus carbonum*, homologous genes for HC-toxin biosynthesis have also been identified in *Alternaria jesenskae* and *Setosphaeria turcica*. However, whether they represent orthologs or paralogs in *S. turcica* has been questioned (Walton 2006; Condon *et al.* 2013; Wight *et al.* 2013). The identification of this putative SM biosynthesis cluster in the *Mvc* genome suggests homology for the production of HC-toxin or an HC-toxin-like compound. *TOXD*, a gene encoding a short-chain alcohol dehydrogenase, has also been previously identified in the *TOX2* locus of *C. carbonum and A. jesenskae* (Walton 2006; Wight *et al.* 2013). An *Mvc* homolog of *TOXD* has also been identified in this study within the HC-toxin-containing SM cluster. Walton (2006) also reported that the *TOX2* locus, which contains the collection of genes responsible for HC-toxin biosynthesis, is loosely clustered over >500kb (Walton 2006). The considerable distance between genes in the *TOX2* locus could provide a plausible explanation for why more *TOX* gene homologs were not identified in *Mvc* by SMURF, as the maximal distance allowed for a gene as part of a cluster was exceeded.

*Mvc* has a complex life cycle involving stages of asexual reproduction and sexual outcrossing. Proteins involved in vegetative cellular fusion and with Het-C domains for heterokaryotic incompatibility were identified in this study, indicating that *Mvc* may be heterokaryotic under conditions that are unfavorable for sexual reproduction. Although two individuals may not be sexually compatible, they may still undergo vegetative hyphal fusion. Sclerotinaceous fungi have been shown to have dynamic mating-type loci, which in some species, allows them to switch between mating types through repeat-induced meiotic sequence deletion within the *MAT* locus (Xu *et al.* 2016). These dynamics may enhance reproductive viability and increase the chances of survival. Abate *et al.* (2018) identified *MAT1-1-1* sequences in *M. fructicola*, *M. fructigena*, and *M. laxa* with high similarity to the direct or inverted repeats responsible for recombination within the *MAT* locus in homothallic members of the Sclerotinia genus (Chitrampalam *et al.* 2013; Xu *et al.* 2016; Abate *et al.* 2018). In these *Monilinia* spp., all *MAT* loci had a heterothallic arrangement, and duplicated copies of this sequence were not present within a given *MAT* locus. In contrast, The *Mvc MAT* locus structure is more consistent with a homothallic structure, although some unusual features were found, suggesting a more complex mating dynamic. In the present study, protein homology analysis revealed two potential endogenous gene fusions that we speculate might be a result of repeat-mediated DNA recombination and/or unequal crossing-over events at the *Mvc*-RL1 *MAT* locus. The first fusion we identified was between mating determinant gene *MAT1-1-1* and *MAT* locus flanking gene *APN2*. The second fusion we identified was between *MAT1-2-10* and *MAT1-1-1*, in which a portion of the 3′ end of *MAT1-1-1* is now located on the 3′ end of *MAT1-2-10*. The *3′MAT1-1-1* translocation and *MAT1-2-10–MAT1-1-1* fusion explains why the *Mvc MAT1-1-1* and *MAT1-2-10* genes are smaller and larger than those of other Sclerotinaceae spp., respectively. Despite the potential recombination-based fusions between *MAT1-1-1–APN2* and *MAT1-2-10–MAT1-1-1* genes, their coding sequences remained in-frame. The 115 bp identical repeat shared between the *tfMAT1-1-1*, and *MAT1-2-*10 genes is homologous with the 146-256 bp repeats present in *Sclerotinia* spp. *MAT* loci that have been shown to facilitate intra-locus recombination events (Chitrampalam *et al.* 2013; Xu *et al.* 2016). We hypothesized that these direct repeats may also facilitate a recombination event that would delete the *MAT1-2-1* and *tfMAT1-2-10* sequences and restore the complete MAT1-1-1 protein alpha 1 domain in-frame. This hypothesis was supported by the identification and the predicted deletion allele in isolate NCWL-11. A second 165 bp direct repeat found in the intragenic regions flanking the *MAT1-1-5* and *tfMAT1-1-1* genes could facilitate a recombination resulting in the deletion of *MAT1-1-5* and *tfMAT1-1-1*. If we presume the sequenced *Mvc*-RL1 isolate to be homothallic, these possible intra-locus recombinations could theoretically produce heterothallic progeny of each mating type depending on which pair of repeat sequence were recombinogenically active. The resulting progeny and progenitors may exist in populations consisting of homothallic and compatible heterothallic isolates. The role these sequences play in the evolution and function of the *MAT* locus will require characterization of the *MAT* locus from other field populations and from direct meiotic progeny. Evidence from field populations indicates that both clonal and outcrossing populations are present in North America (Burchhardt and Cubeta 2015). Research to determine how the complexity of the truncated-fused mating-type genes and the potential for repeat-mediated mating-type recombination influence mating compatibility and population diversity will aid our understanding of pathogen adaptation at the host species and cultivar levels.

In conclusion, this study characterized the putative effectors that *Mvc* may utilize to interact with its host during mummy berry disease infection. This study also characterized the structure of the *Mvc* RL1 and the NCWL-11 *MAT* locus, revealed a complex array of repetitive sequences within the *MAT* locus, and provided genomic evidence of potential heterokaryosis. Genes within the syntenic regions between *Mvc* and close relatives, within the *MAT* locus, and encoding effector proteins could be targeted for disease management strategies.
